# Cardiovascular Disease Risk Factor Patterns and Their Implications for Intervention Strategies in Vietnam

**DOI:** 10.1155/2012/560397

**Published:** 2012-03-04

**Authors:** Quang Ngoc Nguyen, Son Thai Pham, Loi Doan Do, Viet Lan Nguyen, Stig Wall, Lars Weinehall, Ruth Bonita, Peter Byass

**Affiliations:** ^1^Department of Cardiology, Hanoi Medical University, 1 Ton-That-Tung Street, Dong-Da District, 10000 Hanoi, Vietnam; ^2^Vietnam National Heart Institute, Bach Mai Hospital, 78 Giai-Phong Avenue, 10000 Hanoi, Vietnam; ^3^Umeå Centre for Global Health Research, Umeå University, 90187 Umeå, Sweden; ^4^School of Population Health, Faculty of Medical and Health Sciences, University of Auckland, Auckland 1142, New Zealand

## Abstract

*Background*. Data on cardiovascular disease risk factors (CVDRFs) in Vietnam are limited. This study explores the prevalence of each CVDRF and how they cluster to evaluate CVDRF burdens and potential prevention strategies. *Methods*. A cross-sectional survey in 2009 (2,130 adults) was done to collect data on behavioural CVDRF, anthropometry and blood pressure, lipidaemia profiles, and oral glucose tolerance tests. Four metabolic CVDRFs (hypertension, dyslipidaemia, diabetes, and obesity) and five behavioural CVDRFs (smoking, excessive alcohol intake, unhealthy diet, physical inactivity, and stress) were analysed to identify their prevalence, cluster patterns, and social predictors. Framingham scores were applied to estimate the global 10-year CVD risks and potential benefits of CVD prevention strategies. *Results*. The age-standardised prevalence of having at least 2/4 metabolic, 2/5 behavioural, or 4/9 major CVDRF was 28%, 27%, 13% in women and 32%, 62%, 34% in men. Within-individual clustering of metabolic factors was more common among older women and in urban areas. High overall CVD risk (≥20% over 10 years) identified 20% of men and 5% of women—especially at higher ages—who had coexisting CVDRF. *Conclusion*. Multiple CVDRFs were common in Vietnamese adults with different clustering patterns across sex/age groups. Tackling any single risk factor would not be efficient.

## 1. Introduction

Myocardial infarction (MI) and stroke are the leading causes of cardiovascular (CVD) morbidity and mortality worldwide, especially in low- and middle-income countries (LMICs) where 80% of the total CVD burden occurs. CVD death rates, already higher in poorer populations, are also rising, as the death rates in many wealthy countries are waning [1–3]. In Vietnam, stroke is the leading cause of death followed by heart disease [4], although mortality from coronary heart disease has recently risen [5].

Findings from INTERHEART [6] and INTERSTROKE [7] studies suggest that a few traditional modifiable risk factors could explain over 90% of the population attributable risk of both MI and stroke. These include hypertension, abnormal lipids, tobacco use, obesity, diabetes mellitus, diets with low intakes of fruits and vegetables, physical inactivity, excessive alcohol intake, and psychosocial factors. Modification of currently known risk factors has the potential to prevent most premature cases of both MI and stroke worldwide, providing that there are differences in the relative importance of each risk factor for stroke or MI between men and women and across different geographic regions or ethnic groups [6–10], due to variations in risk factor profile, CVD burden, and socioeconomic cultural circumstances. In offering an evidence-based context for policy planners and health education programmes in a low-resource setting like Vietnam, it is important to quantify the proportion of the population at high overall risk of CVD in order to match this with availability of resources. In reality, a substantial proportion of the population carry individual clusters of several risk factors [11], which demonstrates the need for comprehensive population-wide strategies and approaches. When treatment decisions are to be made concerning individual clinical interventions, it is clear that a smaller proportion of people are at highest risk due to individual clustering of risk factors, including age and sex, and need to be identified for rational resource and health system planning.

 This study aims to describe the prevalence of each important CVD risk factor as well as providing a profile of the individual clustering of major CVD risk factors in a representative sample of the adult population of Vietnam, highlighting the differences between men and women. The study also aims to estimate the prevalence of people having high overall 10-year CVD risks using the Framingham general cardiovascular risk score [12]. These findings will be important for optimizing the selection of risk-factor targets for population-based or individual-based programmes to prevent and reduce the burden of cardiovascular diseases in the studied communities as well as in extrapolations to the population of Vietnam.

## 2. Materials and Methods

### 2.1. Study Population and Study Design

A cross-sectional survey was conducted in March and August 2009, using a multistage sampling strategy to identify the prevalence of major cardiovascular risk factors including lipidaemia profile in Thai Binh (a rural province) and Hanoi (a urban province) of Vietnam. This survey followed the framework of the national survey on hypertension, in which Hanoi represented city areas and Thai Binh represented lowland areas, but the blood tests were only taken from a 1-in-5 sample of participants in the city area for fasting glucosaemia and lipidaemia profile due to limited financial resources [13]. Similarly to the previous national survey, a representative sample of the adult population (≥25 years old) from both Hanoi and Thai Binh provinces was randomly selected from 24 primary sampling units (communes: 110 person sample per commune), following 3 communes per district and 4 districts per province [13].

Data were collected at local health stations in the selected communes by trained and qualified surveyors using a questionnaire which included personal medical history of any relevant chronic diseases, demographic background (age, sex, residential area, occupation, and education level) and self-reported behavioural risk factors (smoking history, alcohol consumption, dietary salt habit, daily fruit and vegetable consumption, level of physical activities, level of stress). In addition, all participants were requested to fast overnight in order to have an oral glucose tolerance (OGT) test and a blood sample for lipid profiles (including total cholesterol, triglyceride, low-density lipoprotein cholesterol LDL-C and high-density lipoprotein HDL-C). Blood samples were collected, stored, and analysed by specialists from the Department of Biochemistry, Bach Mai Hospital Hanoi, Vietnam. People with no history of diabetes were asked to perform OGT test loaded with 75 g anhydrous glucose. Portable glucometer devices from Terumo with corresponding strips were used to measure glucosaemia pretest and 2 h after OGT test.

Among 2,640 invited subjects, 2,306 participated in the survey, giving an overall response rate of 87.3% (99.8% in Thai Binh province and 75.0% in Hanoi province). A further 176 (7.6%) participants were excluded from analysis due to pregnancy status or missing important information or blood test results.

### 2.2. Social and Cardiovascular Risk Factors: Assessments and Classification

Occupational status was classified into three groups: government staff, manual workers (farmers, building workers), and other occupations (housewives, handicraft makers, jobless, disabled). Educational level, which was determined by years of schooling and level at graduation, was classified into 2 groups: incomplete secondary schooling (≤9 years of education) and higher (>9 years of education including graduation from high school or higher). Residential area, which was divided into urban and rural, was identified on an administrative basis for each commune within each province.

People who smoked tobacco products such as cigarettes, cigars, or pipes over the previous month were classified as current smokers. People who took more than 2 standard units of drink per day (women) or more than 3 per day (men) were defined as having an excessive alcohol intake. People who ate less than five servings of fruit and/or vegetables on average per day were defined as having a diet with low fruit and vegetable consumption [14]. People who preferred daily foods that contained more salt than the similar foods ordered by other adult members in the family or people around them were classified as having salty diets. Energy requirement in metabolic equivalents (METs) for each individual was estimated based on details of duration and type of all self-reported physical activities in a typical week. People with total physical activity less than 3000 METs minutes per week were classified as physically inactive [15]. Similarly to the INTERHEART study [16], psychosocial stress was assessed and semiquantitated by several simple questions to evaluate whether the participants had any stress at work or at home, any financial stress, any major life events (such as marital separation or divorce, loss of crop or job, major intrafamily conflict, death, illness of a close family member/spouse, etc.) or any other major stress in the past year at different levels (none, mild, moderate, and severe). People who had more than 2 moderate stressors were classified as having psychosocial stress.

Blood pressure (BP) was measured at least twice, at least two minutes apart in a resting and sitting position using an automatic digital sphygmomanometer (OMRON Healthcare Inc., Bannockburn, Illinois, USA), with an appropriate sized cuff, following a similar standardized protocol as undertaken in the national survey. A third measurement was performed if the difference between the first two measurements was more than 10 mmHg. Hypertension was defined as an average systolic BP (SBP) ≥ 140 mmHg, and/or average diastolic BP (DBP) ≥ 90 mmHg, and/or self-reported current treatment with antihypertensive medications [17–20].

Body weight, height, waist and hip circumference were measured by trained and qualified surveyors twice strictly following the standardised protocol previously described elsewhere [13]. Body mass index (BMI) was calculated as weight (kg) divided by height squared (m^2^). Overweight was defined as BMI ≥ 23 and obesity was defined as BMI ≥ 25 or having central obesity (BMI ≥ 23 with waist circumference either ≥90 cm in men or ≥80 cm in women), both mentioned criteria having been specified for South-Asian populations by WHO Regional Office for Western Pacific (WPRO) [21].

Dyslipidaemia was defined as self-reported current treatment with cholesterol-lowering medications and/or having one or more of the following, based on blood test results: total cholesterol ≥5.17 mmol/L; HDL-C <1.03 mmol/L; LDL-C ≥3.36 mmol/L; triglyceride ≥1.7 mmol/L, as recommended by National Cholesterol Education Program (NCEP) Expert Panel on Detection, Evaluation, and Treatment of High Blood Cholesterol in Adults (ATP III) guidelines [22].

Diabetes was defined as fasting glucose ≥7.0 mmol/L and/or 2 h after OGTT glucose ≥11.1 mmol/L and/or self-reported as currently taking any diabetes medication, as recommended by American Diabetes Association (ADA) guidelines [23–26].

### 2.3. Data Analysis

The prevalence of each risk factor and their clustering within individuals were calculated for men and women, stratified by age group to identify the differences in CVD risk factor patterns between women and men. Details of age distribution by sex in urban and rural areas of selected districts in Hanoi and Thai Binh provinces from Vietnam Population and Housing Census in 2009 [27] were used to weight and age-standardise the above prevalences for the studied population as well as for extrapolation to the whole population.

These CVD risk factors were divided into two groups: metabolic factors (including hypertension, abnormal lipids, obesity, diabetes mellitus) and behavioural factors (including tobacco use, excessive alcohol intake, unhealthy diet, physical inactivity, and psychosocial factors). Unhealthy diet was determined from both self-reported diet-related risk factors (either high salt or low fruit and vegetable consumption). People who had ≥2/4 metabolic factors, ≥2/5 behavioural factors, or ≥4/9 of all mentioned risk factors were considered to have individual clusters of respective risk factors.

Framingham general cardiovascular risk scores [12], which apply to individuals from 30 to 74 years old without baseline CVD, were used to estimate the overall 10-year risk of developing coronary heart disease (myocardial infarction, coronary death) and other important potential adverse cardiac events (stroke, heart failure) in the community. The score incorporated the following variables: age, sex, tobacco use, treated and untreated systolic blood pressure, diabetes, and lipid profile (total cholesterol, HDL-cholesterol) or BMI (replacing lipids in a simpler model). People who had overall 10-year cardiovascular risk ≥20% were classified as having a high overall CVD risk.

Both descriptive and analytical statistical analyses were carried out using STATA 11 software (Stata Corporation, Texas, USA). Means with standard errors and proportions with 95% confidence intervals (CIs) for variables of interest were calculated. Multivariable logistic regression analyses were performed to examine the association between social characteristics and clustering of risk factors and their associated odds ratios (ORs) and 95% CIs were presented, separately for women and men. A *P*  value < 0.05 (two tailed) was considered to represent statistical significance.

### 2.4. Ethical Issues

This study protocol was approved by both Scientific Ethical Committees in Biomedical Research at Bach Mai Hospital, Hanoi, Vietnam and at the International Medical Centre of Japan (IMCJ) Hospital, Tokyo, Japan. All human subjects in the study were asked for their consent before collection of data and venous blood, and all had complete rights to withdraw from the study at any time without any threat or disadvantage. Any participants with high blood pressure or other disorders were referred to appropriate facilities for further investigation and treatment.

## 3. Results

After excluding 176 records with missing data, a total of 2,130 subjects were analysed, of which 1,345 (63.2%) were women and 830 (36.5%) were men. The average age for women was 52.0 years and for men 53.7 years; there was no difference in age group structure. The sex ratio in our study population was quite similar to the results from the previous national survey on hypertension [13], in which the study sample was also randomly selected from to the entire list of current inhabitants at the study regions in multistage sampling. Both our study and the previous national survey probably reflected the contemporary sex ratio of the local remaining adult population, which obviously excluded a substantial number of people (mostly male) who temporarily out-migrated to earn money for their families.  Table 1 shows the characteristics of the studied population, including social factors, biological and self-reported behavioural factors. Compared to biological characteristics among women, men had significantly higher weight, waist circumference, waist hip ratio, blood pressure (both systolic and diastolic), LDL-cholesterol, triglyceride, and fasting glucosaemia but lower HDL-cholesterol. There was no difference in BMI and total cholesterol between the sexes. In terms of behavioural risk factors, significantly higher proportions of men were currently smoking (*P* < 0.01), having excessive alcohol intake (*P* < 0.01), unhealthy diet with low consumption of fruit/vegetable or high salt diets (*P* < 0.05), but there were no differences in the proportions of physical inactivity or experience of stress in men compared to women (Table 1). The prevalence of unhealthy diets was lower in women (53%) than in men (60%).

**Figure 1:**
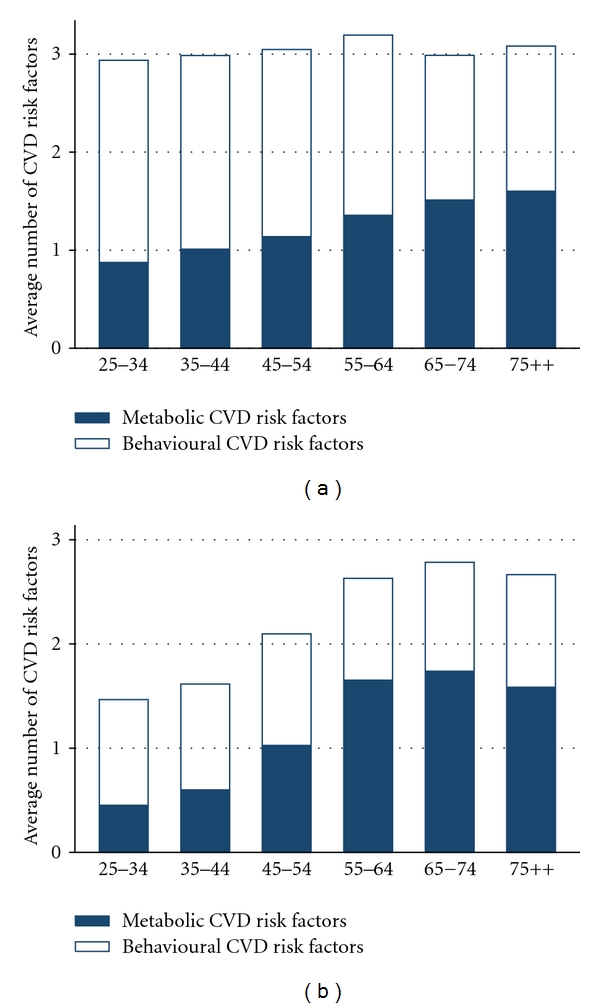
Average number of cardiovascular disease risk factors among men (a) and women (b), stratified by age group.

Table 2 shows the prevalence of each CVD risk factor and prevalence for having clusters of CVD risk factors, stratified by age group and sex, after weighting with the national age distribution in 2009 [27] in order to reflect the current profile of CVD risk factors in the studied population of Vietnam. Overall, the prevalence of all CVD risk factors, except for physical inactivity and experiencing stress, was considerably higher in men than in women. Figures 1(a) and 1(b) show the different trends of clustered CVD risk factors between men and women: the average number of CVD metabolic risk factors in women tended to increase more steeply with age and exceed the trend in men over 55 years of age, while the average number of CVD behavioural risk factors in men tended to decrease with age.

Both versions of Framingham general CVD risk score, one using lipid profiles and the other using BMI, were applied to calculate the overall risk of cardiovascular events within 10 years. Within the studied population, the risks estimated using BMI were higher, around 10% in women and 20% in men, than the estimates using lipid profiles. The prevalence for having an overall risk greater than 15% and 20%, respectively, is shown in Table 3. The prevalence of having high overall CVD risk sharply increased with age, exceeding 10% after the age of 45 years in men and after 55 years in women. 

 Multivariable logistic regression models were constructed to analyse the associations between having clusters of CVD risk factor and age, residence, occupation, and educational level (Table 4). The models showed that having clusters of metabolic risk factors was less common at younger ages, among people living in rural areas or doing manual work for both sexes, while having cluster of behavioural risks was more common in women with higher educational levels and in men with manual jobs. This could be explained by the higher proportion of excessive alcohol intake and physical inactivity in women having higher education or higher proportions of smoking, self-reported unhealthy diet and physical activity in men having manual jobs, while there was no difference among the remaining behavioural factors.

## 4. Discussion

Findings from our study showed that major modifiable CVD risk factors were common and often individually clustered in the studied adult population of Vietnam, increasing with age and having different patterns between sexes. We acknowledge that the cross-sectional design might introduce some misclassification due to self-reported information and the data might not truly reflect the time and context-bound aspects of CVD risk factor patterns. In addition, some factors such as experiencing stress were challenging to measure and there was no clear evidence on how to address stress in primary prevention [28]. Using the same frameworks as the previous national survey and implementing in two similar provinces (Hanoi and Thai Binh) [13], both glucosaemia and lipidaemia disorders were extensively investigated in this study in order to fill gaps in our understanding of major metabolic CVD risk factors in the Vietnamese population, although the data were only available from two provinces rather than the eight provinces in the national surveys, due to limited financial resources. Bearing in mind these limitations, the study tried to obtain a snapshot across a panorama of nine changeable risk factors, which accounted for over 90% risk of cardiovascular events [6, 7], then extrapolating and proceeding to image the contemporary population burden of CVD risk factors both as single factors and within-individual clusters.

Hypertension, smoking, and excessive alcohol intake are considered as the most prominent risk factors for chronic and cardiovascular diseases [13, 29]. Estimates from our study that 26.4% of adults (≥25 years old) suffered from hypertension would extrapolate to 12.5 million people nationally, while only 26.7% (equivalent to 3.3 million) of these hypertensives were treated. However our results showed that lipid abnormalities (60% in the sample, extrapolating to 28.5 million people) and unhealthy diet (54.6%, 25.9 million) were the most common in both sexes, while smoking and excessive alcohol intake were prominent only in men. Future intervention programmes to cover newly emerging CVD risk factors such as unhealthy diets or dyslipidaemia measured by changes in cholesterol levels may be important in countries such as Vietnam where changes in food consumption patterns are occurring at a rapid pace.

Although our data from one cross-sectional survey could not differentiate the sequence in which metabolic risk factors developed, the increasing trend with age for each risk factor was consistent with suggestions that high adiposity and cholesterol often preceded the development of hypertension and diabetes from young adulthood to middle age in 20-year followup of CARDIA study [30, 31], and consistent with causal web of lifestyle risk factors for chronic disease prevention [32].

**Figure 2:**
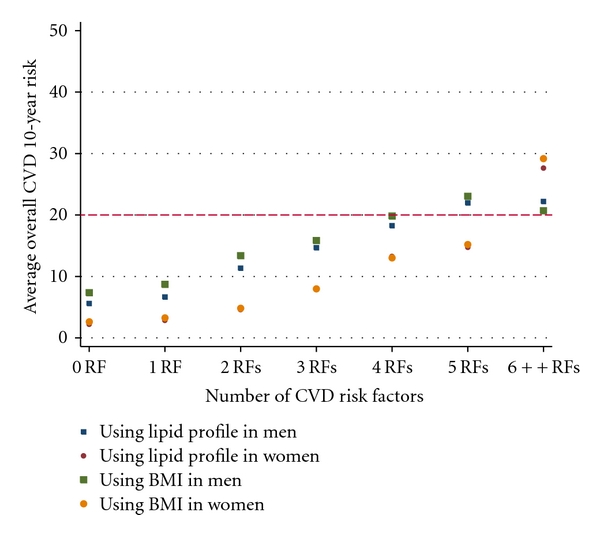
Trends of average overall cardiovascular disease risk by the number of risk factors.

Quite a few studies showed the substantial proportion of CVD risk factors clustered among individuals in the population although the variations could be influenced by various differences in geographical, socioeconomic characteristics, age structure, time of study (seasonal variations), cut-off points for high risk classification, exclusion or inclusion criteria for CVD risk factors [10, 33–35]. Projected from our study, 20.4% adults aged 25 years and above in the population had clusters (≥4/9) of all major CVD risk factors. CVD incidence and mortality increase as quality of life decreases progressively with the number of CVD risk factors [36–39]. In our study, both systolic and diastolic BP increased with the number of risk factors in both sexes. The overall CVD 10-year risk also increased with the number of CVD risk factors in both sexes (Figure 2). In reality, blood pressure control worsened as the number of CVD risk factors increased [40] even with multiple drug therapies [41–43]; therefore, decisions about hypertension management should always consider the presence of other CVD risk factors rather than BP level alone [44].

CVD risk was influenced in a cumulative fashion by socioeconomic, behavioural, and biological factors acting throughout the life course, in which people with lower social economic status would be more susceptible and likely to have CVD risk factors, leading to cardiovascular events later in life [45–50]. Influences on metabolic disorders from lifestyle and culture habits are even stronger than those from genetic factors [51]. Our results suggested the importance of urban living conditions where people had higher prevalence of metabolic disorders after adjusting for age and other social factors, in accordance with results from other studies [8, 33, 52].

 A number of multivariate risk models [12, 22, 53–57] have been developed to integrate individual factors in apparently healthy, asymptomatic individuals for estimating the risk of specific cardiovascular events such as coronary heart disease (fatal or nonfatal) and stroke over a certain period of time. Theoretically, the estimated risk of important cardiovascular events would be very useful both for patient education (e.g., motivating patients to adhere to risk-reduction therapies) and for clinical practice (identification of high-risk patients who deserved immediate care and modification of the intensity of management strategies). However, the complexity of the equation, time and context-bound results, confused assessment of outcome or risk factors, lack of some variables in low-resource settings, regular need for validation [58, 59], regulatory constraints, and the nature of the physician-patient relationship [60] all are hidden barriers to the routine use of CVD risk scores in daily practice, especially in primary care where blood tests were not available in low-resource settings. In addition, the overall risk stratification approach is likely to counter the established clinical practice in most LMICs that tend to focus on risk-factor thresholds, even though risk-based care is more effective and cost-effective [61]. 

 In this study, the Framingham general cardiovascular 10-year risk scores [12] were applied to estimate potential adverse cardiovascular events individually and then totally in the studied population, including both stroke and coronary heart disease outcomes, bearing in mind that these scores could be overestimates or underestimates of the event risks in the population of Vietnam, where there has been no validation or calibration studies so far. We acknowledge that the equation only covered a few CVD risk factors, and their impacts on predicted outcomes were assumed to be linear for all variables and similar to the original Framingham population, which might not be true in the context of transition and development in contemporary Vietnam. Bearing in mind these limitations, an estimate of 10% in the studied population (extrapolated to 3.7 million people in the Vietnamese population) aged from 30 to 74 (4.6% in women and 20.4% in men) had an overall CVD 10-year risk ≥20%; the more risk factors, the higher the overall CVD risk [33]. The results also showed the homogeneity between two versions of the Framingham score using either BMI or lipid profiles (Table 3) and suggested that the simplified score version using BMI has potential advantage for wider application in low-resource settings, obviating the need for blood tests for lipid profiles in prioritising available strategies or approaches to intervention against CVD risk factors in primary care. Absolute risk charts using similar predictors (age, sex, smoking status, SBP, BMI, and/or diabetes) were a feasible and replaceable solution [61] for individuals in daily practice but were not sensitive enough to capture small changes in overall risk resulting from interventions and for summarising the benefits for heterogeneous populations with diverse CVD risk patterns. However, further cohort studies should be used to calibrate these equations in order to improve the local predictability of future cardiac events.

**Figure 3:**
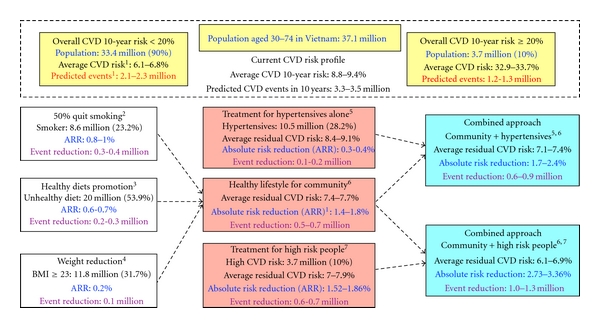
Estimation of cardiovascular burden and potential benefits of intervention strategies for the adult population of Vietnam, extrapolated from the average of individual overall CVD 10-year risks in a studied population. ^1^ Overall cardiovascular (CVD) risk, residual risk, absolute risk reduction (ARR), and predicted CVD events or predicted event reduction were estimated by both versions of Framingham general risk score, one used lipid profile and the other used BMI, and weighted by national age structure of the Vietnamese population in 2009. ^2^ In assumption that the prevalence of current smoking will reduce by 50%. ^3^ In assumption that the effect of healthy diet (especially salt reduction) will reduce 5 mmHg of systolic blood pressure (SBP). ^4^ In assumption that the obesity (BMI ≥ 23) will reduce 10% of weight, the risk was only estimated by BMI version of Framingham general risk score. ^5^Approach for hypertensive alone included drug therapy to control BP (targeted SPB ≤ 140 for any hypertensives and ≤130 for diabetes). ^6^Approach for community included healthy lifestyle promotion campaigns: quitting smoking (in assumption of 50% reduction of current prevalence), healthy diet (salt reducuon, low-fat and high-fiber diet, in assumption of 5 mmHg reduction of SBP), and encouraging physical activity and 10% weight reduction for obesity (BMI ≥ 23). ^7^ Approach for high-risk people (overall CVD 10-year risk ≥20%) included quitting smoking (100%), drug therapy to control BP (targeted SBP ≤ 140 for any hypertensives and ≤130 for diabetes), statin for dyslipidaemia (in assumption of 20% reduction of total cholesterol, 10% increase HLD-C), and 10% weight reduction for obesity (BMI ≥ 23).

Based on individual calculated overall risk profiles, we estimated the average overall risk at the population level and predicted potential adverse cardiovascular events over 10 years. Our extrapolations revealed that the average overall risk for any cardiovascular event over 10 years for whole population aged from 30 to 74 years was 8.8–9.4%, in other words, 3.3–3.5 million CVD events could happen over 10 years (Figure 3). It has been estimated that just three cost-effective interventions, tobacco control, salt reduction, and a multidrug clinical service to treat individuals at high overall risk of cardiovascular disease would avert deaths in Vietnam [61, 62]. Recently, other interventions, though less cost-effective and feasible, have been implemented to tackle unhealthy diets, physical inactivity, obesity [63], focusing more on BMI-mediated distal risk factors [32] as well as policy-level solutions to create favourable environments for implementing effective strategies in primary care [64]. Based on some assumptions about the effectiveness of healthy lifestyle interventions [65–69] or drug therapy to manage blood pressure [19] or dyslipidaemia [22], we tried to calculate the absolute risk reduction (ARR) of average overall CVD risk in the population and predict the reduction of potential adverse cardiovascular events, which could arise as benefits from various scenarios of risk factor intervention (Figure 3).

Previous studies showed that hypertension is a major public health problem in Vietnam [13, 29], requiring a lot of effort to detect and deliver appropriate management, constituting a high priority in the existing system of primary care. However, our extrapolated estimation suggested that treatment of a CVD risk factor alone (such as hypertension) without taking into consideration other modifiable CVD risk factors (such as smoking, unhealthy diet) would not be an efficient approach for achieving a high general health impact. A population strategy to reduce tobacco consumption in men and halt the rise in women should be the first priority. The high level of unhealthy diet and potential benefit from interventions suggests a population-wide strategy though the mass media aimed at reducing salt content in food is the next strategy. The high-risk individual approach would benefit the entire population more than only approaching hypertensives. If there were not enough resources to assess overall CVD risk on a wide scale, especially where expensive blood tests are required, simplified equations using age, sex, tobacco use, blood pressure levels, and BMI could be used to estimate the overall risk [61]. In addition, where resources allow, a combined community approach (mostly by healthy lifestyle promotion) and individual approaches using simpler and more feasible measurements to identify people at high risk could be employed.

## 5. Conclusions

In conclusion, nine major CVD risk factors, often clustered within individuals, were common in the adult population of Vietnam with differences noted between sex and age groups, testifying to the need for inclusion of age and sex in any risk prediction models. Tackling any single risk factor alone without considering other modifiable CVD risk factors is not an efficient or sustainable approach. Combination of population and individual approaches are required to reduce the burden of CVD risk factors and maximise the protective effects for the whole community. Modification and calibration of an existing score for the Vietnamese population, for identifying individuals at high risk of CVD, is a priority.

## Figures and Tables

**Table 1:** General characteristics of the study population.

Characteristics	Women (*n* = 1, 345)	Men (*n* = 785)
Biological factors	Mean ± SD	Mean ± SD

Age (year)	52.0 ± 14.3	53.7 ± 14.7
Weight (kg)	49.6 ± 8.1	56.2 ± 9.4
Body mass index BMI (kg/m^2^)	21.5 ± 3.1	21.5 ± 3.0
Waist circumference (cm)	73.5 ± 7.5	75.9 ± 7.9
Waist-hip ratio	0.85 ± 0.06	0.88 ± 0.06
Systolic blood pressure (mmHg)	129.1 ± 23.0	135.0 ± 22.0
Diastolic blood pressure (mmHg)	77.3 ± 12.0	80.4 ± 12.4
Total cholesterol (mmol/L)	4.69 ± 0.99	4.65 ± 1.06
HDL cholesterol (mmol/L)	1.32 ± 0.33	1.26 ± 0.34
LDL cholesterol (mmol/L)	2.71 ± 0.73	2.59 ± 0.75
Triglyceride (mmol/L)	1.80 ± 1.29	2.19 ± 1.82
Fasting glucosaemia (mmol/L)	4.7 ± 1.2	5.0 ± 1.3
OGTT-2 h glucosaemia (mmol/L)	6.8 ± 2.2	6.8 ± 3.0

Self-reported behavioural factors	%	%

Current daily smoking	4.3	54.1
Excessive alcohol intake	1.1	24.1
Low fruit and vegetable diet	38.1	44.3
High salt diet	27.1	32.2
Physical inactivity	11.5	13.7
Having stress	25.3	22.2

Social factors	%	%

*Residence *		
In rural area	51.9	45.7
In urban area	48.1	54.3
*Education level *		
Secondary school and below	69.3	67.5
High school and above	30.7	32.5
*Occupation *		
Government staff	18.7	24.0
Manual workers	60.5	64.7
Other	20.8	11.3

**Table 2:** Prevalence of cardiovascular diseases risk factors in a studied population of Vietnamese adults stratified by sex and age group.

Major cardiovascular disease (CVD) risk factors	Prevalence in women by age group (%)						Prevalence in men by age group (%)						Prevalence by sex (%)	
	25–34	35–44	45–54	55–64	65–74	≥75	25–34	35–44	45–54	55–64	65–74	≥75	Women	Men
*Metabolic CVD risk factors*														
Hypertension	4.4	7.2	25.4	50.4	63.1	63.5	12.4	22.2	31.4	43.9	61.7	66.2	25.0^a^	31.2
Diabetes	0.0	4.5	5.4	13.2	12.5	13.7	3.2	4.8	9.8	9.6	12.4	21.4	6.2^a^	8.0
Obesity	8.0	11.1	18.6	29.3	27.6	19.1	19.8	11.6	11.6	18.2	16.5	10.8	17.4^b^	14.5
Dyslipidaemia	33.4	38.0	56.0	74.7	72.0	66.5	54.8	65.5	63.5	66.9	61.8	63.9	52.4^b^	62.8

*Behavioural risk factors*														
Current smoking	2.5	6.0	2.2	4.5	6.4	3.5	56.1	65.4	61.7	58.7	44.5	25.3	3.8^a^	58.8
Excessive alcohol intake	1.2	0.6	0.6	1.6	1.7	0.0	27.9	31.6	30.8	22.8	17.8	8.1	0.9^a^	27.6
Unhealthy diet	52.8	52.2	52.7	49.3	47.8	62.2	70.3	53.2	57.2	62.0	52.7	66.3	52.0^a^	59.4
Physical inactivity	20.4	16.5	19.9	18.2	24.4	27.1	26.1	19.1	19.2	15.6	20.7	32.4	19.3	20.3
Having stress	24.5	26.4	31.5	24.1	24.2	15.2	25.8	28.3	22.0	24.6	11.8	16.0	27.1	23.5

*Individual clustering of CVD risk factors*														
≥2/4 metabolic risk factors	9.8	14.4	26.1	54.5	60.7	51.1	24.9	24.9	30.6	41.7	48.8	57.1	28.1	32.1
≥2/5 behavioural risk factors	26.7	22.5	31.0	24.7	24.9	32.4	64.4	64.5	63.1	60.4	49.9	51.9	27.0^a^	62.0
≥4/9 major CVD risk factors	4.8	4.3	15.3	21.6	28.3	23.2	30.4	33.5	35.7	35.1	36.2	42.5	13.0^a^	34.4

^a^ *P* < 0.01; ^b^ *P* < 0.05 when compared between men and women.

**Table 3:** Average estimated overall CVD 10-year risk using Framingham general risk score (either using lipid profile or BMI) and prevalence of high overall risk in a studied population of Vietnamese adults, stratified by sex and age group.

	Average overall risk (%)		Difference (%) between (1) and (2)	Prevalence of overall (%)		
	Using lipid profile (1)	Using BMI (2)		Risk ≥10%	Risk ≥20%	Risk ≥30%
*Women*						
30–34	1.0	1.1	7.3	0.0	0.0	0.0
35–44	1.9	2.0	11.9	0.0	0.0	0.0
45–54	5.1	5.3	10.9	5.9	1.3	0.1
55–64	12.0	12.3	7.0	41.2	13.6	4.2
65–74	17.1	18.0	11.0	68.4	27.5	9.3

*Men*						
30–34	3.3	3.6	23.2	0.0	0.0	0.0
35–44	7.1	7.3	19.5	14.3	0.8	0.0
45–54	13.7	15.5	21.1	63.7	12.9	1.6
55–64	22.7	25.0	19.7	86.9	46.9	20.4
65–74	37.0	39.6	12.9	98.4	81.4	57.2

*Total*						
Women	5.8	6.1	10.1	13.9	4.6	1.4
Male	14.6	16.0	20.0	52.5	20.4	9.0
Both sexes	8.8	9.4	13.4	27.0	10.0	3.9

**Table 4:** Adjusted odds ratios (OR) with 95% confidence interval (CI) for having individually clustered CVD risk factors in a studied population of Vietnamese adults.

Social factors	Having cluster (≥2/4) of metabolic CVD risk factor		Having cluster (≥2/5) of behavioural CVD risk factor		Having cluster (≥4/9) of all major CVD risk factor	
	Women OR (95% CI)	Men OR (95% CI)	Women OR (95% CI)	Men OR (95% CI)	Women OR (95% CI)	Men OR (95% CI)
*Age group*						
25–34	1	1	1	1	1	1
35–44	1.9 (0.9–3.8)	1.3 (0.7–2.6)	1.0 (0.6–1.5)	0.8 (0.4–1.4)	1.2 (0.4–3.7)	1.2 (0.7–2.2)
45–54	3.4 (1.8–6.6)^a^	1.9 (1.0–3.6)^b^	1.5 (1.0–2.3)	0.7 (0.4–1.2)	4.5 (1.8–11.7)^a^	1.2 (0.7–2.0)
55 – 64	12.8 (6.7–24.5)^a^	3.0 (1.6–5.6)^a^	1.0 (0.6–1.6)	0.7 (0.4–1.1)	6.8 (2.6–17.5)^a^	1.2 (0.7–2.1)
65 – 74	16.5 (8.3–32.9)^a^	3.2 (1.7–6.2)^a^	1.0 (0.6–1.6)	0.5 (0.3–0.8)^b^	9.8 (3.7–26.9)^a^	1.3 (0.7–2.2)
75 ++	14.0 (6.8–29.1)^a^	4.4 (2.2–9.2)^a^	1.3 (0.8–2.4)	0.5 (0.2–0.9)^b^	9.8 (3.5–27.0)^a^	1.4 (0.7–2.7)

*Residence area*						
Rural	1	1	1	1	1	1
Urban	2.6 (1.9–3.5)^a^	1.9 (1.4–2.7)^a^	0.8 (0.6–1.1)	0.9 (0.7–1.3)	2.9 (1.9–4.3)^a^	1.8 (1.3–2.5)^a^

*Educational status*						
High school and higher	1	1	1	1	1	1
Less than high school	0.9 (0.7–1.2)	0.9 (0.6–1.3)	0.7 (0.6–1.0)^b^	1.0 (0.7–1.4)	0.8 (0.6–1.1)	0.9 (0.6–1.3)

*Occupational status*						
Manual workers	1	1	1	1	1	1
Government staff	1.0 (0.7–1.5)	2.0 (1.3–2.9)^a^	0.8 (0.5–1.1)	0.7 (0.5–1.0)^b^	1.0 (0.6–1.5)	0.9 (0.6–1.4)
Others	1.4 (1.0–2.0)^b^	1.3 (0.8–2.1)	0.8 (0.6–1.2)	0.9 (0.5–1.4)	1.1 (0.7–1.6)	0.9 (0.6–1.5)

^a^ *P* < 0.01; ^b^ *P* < 0.05.
